# The connection between anemia and limitations in daily activities among older males: the critical role of dynapenia

**DOI:** 10.1007/s40520-024-02859-8

**Published:** 2024-10-26

**Authors:** Abdulkadir Karismaz, Pinar Soysal, Rafet Eren, Istemi Serin, Ceyda Aslan, Masoud Rahmati, Dong Keon Yon, Lee Smith

**Affiliations:** 1 Department of Hematology, University of Health Sciences, Istanbul Training and Research Hospital, İstanbul, Turkiye Turkey; 2 Department of Geriatric Medicine, Faculty of Medicine, Bezmialem Vakif University, Istanbul, Turkiye Turkey; 3 Department of Hematology, Faculty of Medicine, Biruni University, Biruni University Hospital, Istanbul, Turkiye Turkey; 4 Department of Hematology, University of Health Sciences, Istanbul Basaksehir Cam and Sakura City Hospital, Istanbul, Turkiye Turkey; 5 Department of Hematology, Istinye University Faculty of Medicine, Liv Hospital, Istanbul, Turkiye Turkey; 6 Department of Physical Education and Sport Sciences, Faculty of Literature and Human Sciences, Lorestan University, Khoramabad, Iran; 7 Center for Digital Health, Medical Science Research Institute, Kyung Hee University Medical Center, Kyung Hee University College of Medicine, Seoul, Republic of Korea; 8 Centre for Health Performance and Wellbeing, Anglia Ruskin University, Cambridge, UK; 9 Department of Physical Education and Sport Sciences, Faculty of Literature and Humanities, Vali-E-Asr University of Rafsanjan, Rafsanjan, Iran; 10 CEReSS-Health Service Research and Quality of Life Center, Assistance Publique-Hopitaux de Marseille, Aix-Marseille University, Marseille, France

**Keywords:** Older men, Anemia, Activities of daily living, Dynapenia

## Abstract

**Aim:**

The aim of the present study was to examine the relationship between anemia and basic and instrumental activities of daily living in older male patients.

**Methods:**

A total of 223 older males attending one geriatric outpatient clinic were included in this cross-sectional study. Anemia was defined as a hemoglobin level below 13 g/dL. Patients’ demographic characteristics, comorbidities, and comprehensive geriatric assessment parameters were also recorded. Handgrip strength of < 27 kg for males was accepted as dynapenia. Basic Activities of Daily Living (BADL) and Instrumental Activities of Daily Living (IADL) questionnaires were used to evaluate functional capacity.

**Results:**

The mean age (standard deviation) of the participants was 80.17 (7.69) years. The prevalence of patients with anemia was 43.9%. There was differences between anemic and non-anemic groups in terms of presence of diabetes mellitus (DM), congestive heart failure (CHF), chronic kidney disease (CKD), malnutrition, dynapenia, geriatric depression, BADL and IADL scores (all *p* < 0.05). In multivariate analysis, after adjusting for all confounding variables except for dynapenia, patients with anemia were associated with reduced BADL and IADL (all *p* < 0.05). After adjusting for all confounding variables including dynapenia, deterioration in total BADL and IADL scores did not remain significant in the anemic group compared to the non-anemic group (*p* > 0.05).

**Conclusion:**

Close to one in two older outpatient men had anemia. Anemic men had a higher incidence of DM, CHF, CKD, malnutrition, geriatric depression and dynapenia. Anemia was associated with dependence in both BADL and IADL in older men. However, comorbidities, nutritional status, depressive mood and, specifically muscle strength, were important contributors to this association.

## Introduction

In the older population, anemia poses a substantial challenge, demonstrating a clear negative effect on the progression of coexisting conditions and longevity [1–3]. The overall prevalence of anemia stands at 17%, with variations across different settings: 12% in community-based studies, 47% in nursing homes, and 40% among hospital admissions [1]. The World Health Organization defines anemia as a condition where blood hemoglobin (Hb) levels fall below 12 g/dl for women and 13 g/dl for men. This condition is frequently observed in people aged 65 and older [4]. The occurrence of anemia tends to increase with advancing age and is slightly more prevalent in males compared to females [4]. Older individuals can develop anemia due to multiple factors. Among these, chronic disease is the most prevalent cause [5–7]. Several conditions more common in older adults, such as chronic infection, inflammatory disease, and malignancy, are linked to anemia caused by chronic disease. In the older population, iron deficiency is common and typically results from acute or chronic blood loss through the gastrointestinal system. In older individuals, additional significant contributors to anemia include deficiencies in folate and cobalamin (vitamin B12), which can result from insufficient intake or decreased absorption [5, 8]. Furthermore, impaired kidney function is frequently observed in older patients [9]. Depending on the studied population, approximately 14–50% of anemia cases in the older have no clear identifiable cause [4, 7].

Anemia serves as a significant indicator of unfavorable outcomes in later life, including increased risk of falls, disability, hospitalization, and death [10, 11]. Research conducted over 4 and 11-year periods revealed that individuals with anemia face a mortality risk exceeding 50% compared to their non-anemic counterparts in older American populations [1, 3]. The elevated mortality rate may be linked to inadequate oxygen delivery to body tissues, potentially leading to organ dysfunction and disruptions in essential bodily processes, thereby increasing the likelihood of death [12, 13]. An increasing number of cross-sectional investigations indicate a connection between anemia and functional outcomes in older individuals, including dynapenia, which is characterized by the age-related reduction in muscle strength [14–16]. Research by Alexandre et al. revealed that anemia is linked to dynapenia in older Brazilian adults, while Cesari et al. discovered that anemic older Italian adults exhibit lower ankle extension strength compared to their non-anemic counterparts [14, 17]. A comparable investigation conducted among older Korean adults revealed that those with anemia had a higher likelihood of experiencing dynapenia compared to their non-anemic counterparts. This relationship was found to be more significant in male participants than in females [18, 19]. Anemia’s impact on reducing oxygen delivery to tissues also impacts musculoskeletal structures, which may explain the diminished strength and muscle function observed in affected individuals [18]. These factors can collectively contribute to a reliance on assistance for daily living activities. However, there is currently no literature illuminating the relationship between anemia and functionality and related factors in older adults.

Anemia can develop due to sex-based disparities. In males, for instance, anemia may be linked to insufficient sex hormone levels; nevertheless, a significant number of cases remain unexplained [20, 21]. Anemia among men has not been given appropriate attention mainly owing to the lower number of the affected proportion when compared to women [22]. Indeed, the importance of anemia has been studied more in older women than in older men [23]. Considering the results mentioned, we hypothesized that there can be a significant relationship between anemia and the ability to perform basic and instrumental activities of daily living in older male patients. We also sought to obtain more accurate estimates by adjusting for potential key confounding factors, including decreased muscle strength [24, 25]. Therefore, this study exclusively focused on older male participants due to the potential influence of hormonal, socioeconomic, and anthropometric variations between genders on the outcomes of anemia development and comprehensive geriatric assessment parameters.

## Methods

### Patients

A total of 223 male patients who presented to one geriatric outpatient clinic in Turkey and underwent comprehensive geriatric assessment (CGA) were included in the study. One geriatrician and a gerontologist completed CGA in 45 min for each patient. All assessments were performed by the same geriatrician. The same team collected the study data.

### Inclusion/exclusion criteria

All male patients aged ≥ 65 years who presented to one geriatric outpatient clinic for any reason were included in the study. Female patients, patients with acute bleeding, including massive hematuria, hematochezia, melena, hematemesis, or intra-abdominal bleeding, patients with moderate and severe dementia, patients who were not able to undergo CGA due to their current clinical condition (e.g. delirium), patients who could not walk with an assistive device, those with localized muscle strength reduction due to stroke, those with severe visual and/or hearing impairment that prevented communication and understanding of commands during examination, those who refused to participate, those with a terminal disease (e.g. cancer), and those who were hospitalized for a life-threatening illness or major surgery in the last 6 months were not included in the study. As a result, 223 patients were available for study analysis.

### Barthel Index for Activities of Daily Living (BADL) and Lawton Instrumental Activities of Daily Living (IADL) scale

The Barthel Index for Activities of Daily Living (BADL) measures the level of independence and functional status in basic daily activities like feeding, bathing, dressing, bowel/bladder control, using the toilet, transfers (from bed to chair), mobility (on flat surfaces), and stair climbing. A score of 100 indicates complete independence, whereas a score of 0 indicates complete dependency on another person [26].

Disability levels and other factors in community-dwelling older adults were first measured and evaluated by the researchers Lawton and Brody in 1969 using the Lawton Instrumental Activities of Daily Living Scale (IADL). The IADL, the most used assessment tool for this outcome in the older, measures eight abilities, including the use of a telephone, shopping, food preparation, housekeeping, laundry, mode of transportation, responsibility for own medication, and ability to handle finances. Each activity is given a score ranging from 0 to between 2 and 4 in the Turkish version. Low scores indicate a high degree of dependency, and the scale runs from 0 to 23 [27].

### Comprehensive geriatric assessment

Patients’ age, education level, and comorbidities including diabetes mellitus (DM), hypertension, coronary artery disease, chronic renal failure (CRF) , chronic obstructive pulmonary disease, cerebrovascular disease, congestive heart failure (CHF), peripheral artery disease, Parkinson’s Disease, and osteoarthritis were recorded. All participants underwent CGA including Standardized Mini-Mental State Examination (MMSE), Geriatric Depression Scale-15 for neurocognitive evaluation, Tinetti Performance-Oriented Assessment of Mobility (POMA) for mobility evaluation. Mini Nutritional Assessment (MNA) was performed in all patients to detect nutritional status. If the total MNA score was < 17, the patient was categorized as malnourished. Depression was diagnosed using the geriatric depression scale-15 (GDS-15). A score of ≥ 5 on the GDS-15 was considered as depression [28]. Calf circumference was assessed in sitting or supine position. For individuals in sitting position, measurements were taken on a chair in 90°-knee flexion with feet resting on the floor or on the footrest in a wheelchair. In the supine position, the knee joint was flexed at 90° with the feet and ankles relaxed. The flexible tape was wrapped perpendicular around the leg axis without pressing the tissue. Measurement was taken at maximum circumference [29].

#### Dynapenia

Handgrip strength was assessed three times on each hand using the Jamar hand-held hydraulic dynamometer (Jamar hand dynamometer; Sammons Preston, Inc., Bolingbrook, IL, USA). Handle width was adjusted to hand size. Individuals were standing with their arms parallel to their trunk and were encouraged to squeeze the dynamometer as hard as possible. The maximal handgrip strength was recorded. A maximal handgrip strength of < 27 kg for males was accepted as dynapenia [30].

### Laboratory findings

Laboratory tests were performed to evaluate inflammatory markers, the biochemical, metabolic, and nutritional status of the patients included complete blood count, kidney and liver functions, thyroid stimulating hormone, HbA1c and iron levels, iron-binding capacity (IBC), ferritin, vitamin B12, folic acid and vitamin D (25-hydroxy D3). Creatinine clearance from the kidney was estimated by the glomerular filtration rate (GFR) calculated using the chronic Renal Disease Epidemiology Collaborative (CKD-EPI) equation. Chronic Renal Failure (CRF) is defined as kidney damage or estimated GFR below 60 ml/min/1.73 m2.

Anemia was defined as hemoglobin concentrations below 13 g/dL.

### Statistical analyses

Descriptive statistics of qualitative variables are presented as numbers and percentages, and the descriptive statistics of the quantitative variables are presented as mean, standard deviation, median, minimum, and maximum. The conformity of the quantitative variables to the normal distribution was evaluated with the Kolmogorov Smirnov test. The Mann Whitney U test was used to compare the mean of two independent groups. Pearson chi-square analysis was used for comparisons between the groups in terms of the ratios of the relevant qualitative variables. Logistic regression analysis was used to evaluate the relationships between the dependent variable and the independent variables. Enter method was used as variable selection method. The statistical significance level was taken as two-sided *P* ≤ 0.05, and the SPSS (version 26.0; IBM Corp, Armonk, NY, USA) package program was used for analyses. It was calculated that at least 198 older male patients needed to participate in the study with a 5% margin of error and a 95% confidence interval.

## Results

Of the 223 older male patients, 43.9% (*n* = 98) had anemia. The mean age of the patients was 80.17 ± 7.69 years. Patient characteristics, comorbidities, laboratory findings and geriatric syndromes/CGA parameters are summarized in Table 1. A significant difference was observed between the anemic and non-anemic groups in terms of the presence of DM, CRF and CHF (*p* < 0.05). There was no difference between the two groups in terms of education, age and other comorbidities (*p* > 0.05) (Table 1).

**Table 1:** Patients’s characteristics according to anemic and non-anemic groups

	Older male		
	Anemia (-)	Anemia (+)	*p*
Age, years	78.6 ± 7.6	82.1 ± 7.4	0.640
Education	7.9 ± 5.3	6.6 ± 4.5	0.083
**Comorbidities (%)**			
Diabetes Mellitus	32	44.9	0.049
Hypertension	70	83	0.357
Coronary Arterial Disease	23.2	35.1	0.052
Chronic obstructive pulmonary disease	10.3	13.6	0.457
Cerebrovascular Disease	8.8	15.5	0.126
Congestive Heart Failure	6.4	17.5	0.009
Pulmonary Arterial Hypertension	4.8	5.2	0.904
Parkinson Disease	4	8.3	0.180
Osteoarthritis	10.4	10.4	0.997
Chronic Renal Failure	29.1	60.2	< 0.001
**Laboratory Analyses (Blood)**			
Hemoglobin (Hb-g/dl)	15.1 ± 8.2	11.4 ± 1.3	< 0.001
Hematocrit (Hct-%)	43.8 ± 3.5	35.9 ± 3.7	< 0.001
Mean Corpuscular Volume (MCV-fL)	90.2 ± 4.2	85.7 ± 7.2	< 0.001
Red Cell Distribution Width (RDW- %)	13 ± 1.3	14 ± 2.2	< 0.001
White Blood Cell (WBC-mm^3^)	7.6 ± 1.8	7.7 ± 2.3	0.872
Mean platelet volüme (MPV-fl)	8.3 ± 1.8	8 ± 1.9	0.280
Iron (Fe-mcg/dL)	95.1 ± 55.4	57.7 ± 29.1	< 0.001
Iron Binding Capacity (IBC-mcg/dL)	258.6 ± 398.6	234 ± 80.7	0.218
Ferritin (ng/mL)	87.1 ± 84.7	132.7 ± 175.1	0.499
Vitamin B12 (pg/mL)	416.4 ± 295	464 ± 361.9	0.781
Folate (ng/mL)	7 ± 3	7.3 ± 4.2	0.574
25-OH-D3 (VitD-ng/mL)	25.1 ± 15.1	23.3 ± 14.5	0.270
Tyroid Stimulating Hormone (mU/L)	1.8 ± 3.5	2.2 ± 3.7	0.184
**Comprehensive Geriatric Assessment/Geriatric Syndromes**			
MNA Total score	25 ± 3.2	22.3 ± 4.9	< 0.001
GDS-15	3.2 ± 3.5	4.4 ± 3.9	0.006
Hand Grip Strength (kg)	29.3 ± 7.7	23.2 ± 7.7	< 0.001
S-MMSE	25.9 ± 2.6	25.8 ± 2.5	0.640
SARC-F score	1.8 ± 2.3	4 ± 3.3	< 0.001
Tinetti balance	14.8 ± 2.9	13.5 ± 3.9	0.003
Tinetti gait	11.1 ± 2.2	10.3 ± 2.8	0.003
Tinetti total	26.1 ± 4.4	23.8 ± 6.4	0.002

MNA: Mini Nutritional Assessment (0 [worst]-30 [best]); GDS-15: Geriatric Depression Scale-15 (0 [best]-15 [worst]); SMMSE: Standardized Mini-Mental State Examination (0 [worst]-30 [best]); BADL: Basic activities of daily living (0 [worst]-100 [best]); IADL: Instrumental activities of daily living (0 [worst]-23 [best]); Frail total score: (0 [best]-5 [worst]); POMA: Tinetti performance oriented mobility assessment (0 [worst]-28 [best])

Numerous laboratory parameters differed between groups with and without anemia. While MCV, iron, IBC and GFR were lower in the group with anemia, red cell distribution width were higher. All CGA parameters were more negatively affected, except for MMSE, and geriatric syndromes were more common in patients with anemia compared to those without anemia (Table 1).

Table 2 shows that after adjusting for age, in those with anemia compared to those without, adverse effects on malnutrition, dynapenia, BADL, IADL, and GDS scores were statistically significant (*p* < 0.05) (Model 2). In Model 3 multivariate analysis, adjustment was made according to age, DM, CHF, and CRF. BADL [OR: 0.95 (95% CI 0.93–0.98)], IADL [OR: 0.91 (95% CI: 0.87–0.97)], dynapenia [OR: 3.44 (95% CI: 1.78–6.66)], and malnutrition [OR: 4.07 (95% CI: 1.14–11.58)] scores were statistically significant (*p* < 0.05). In Model 4, in addition to the confounders in Model 3, GDS and malnutrition were added to the adjustment, and the significant association of anemia with deterioration in BADL and IADL scores remained (*p* < 0.05) (see Table 2). However, after adjustment Model 4 + Dynapenia, there was no significant relationship between anemia and ADL’s.

**Table 2:** Multiple logistic regression analysis of the relationship between geriatric assessment parameters/geriatric syndromes and anemia

Parameters	Model1		Model2		Model3		Model4	
	OR	*P*	OR	*P*	OR	*P*	OR	*P*
Age	1.06(1.02–1.10)	0.001	-	-	-	-	-	
DM	1.73(1.00-2.99)	0.049	2.16(1.20–3.87)	0.009	-	-	-	
CHF	3.10(1.28–7.54)	0.012	2.88(1.17–7.10)	0.021	-	-	-	
CRF	3.69(2.07–6.57)	< 0.001	3.16(1.75–5.71)	< 0.001	-	-	-	-
Malnutrition	3.94(1.36–11.38)	0.011	3.80(1.27–11.27)	0.016	4.07(1.14–11.58)	0.031	-	-
Dynapenia	3.94(2.25–6.90	< 0.001	3.38(1.85–6.179	< 0.001	3.44(1.78–6.664	< 0.001	3.26(1.63–6.49)	0.001
GDS	1.10(1.02–1.18)	0.013	1.09(1.01–1.18)	0.023	1.07(0.98–1.16)	0.105	-	-
Barthel score	0.94(0.91–0.97)	< 0.001	0.46 (0.92–0.97	< 0.001	0.95(0.93–0.98)	0.001	0.96(0.93–0.99	0.021*
Lawton score	0.88 (0.84–0.93	< 0.001	0.89(0.85–0.94)	< 0.001	0.91(0.87–0.97)	0.002	0.93(0.88–0.99)	0.037*

OR, Odds ratio; *p* values were still significant, even after adjustment for age, DM, HT, CAD, CRF, MMSE and malnutrition

DM: Diabetes Mellitus; HT: Hypertension; CHF: Congestive Heart Failure; CRF: Chronic Renal Failure; GDS: Geriatric Depression Scale

Table 3 shows that after adjusting for age, DM, CHF and CRF, in those with anemia compared to those without, adverse effects on feeding [OR: 2.74 (95% CI: 1.18–6.36)], bathing [OR: 1.34 (95% CI: 1.01–1.76)], dressing [OR: 1.57 (95% CI: 1.02–2.42)], mobility [OR: 0.95 (95% CI: 0.91–0.98)], and stair climbing [OR: 1.49 (95% CI: 1.13–1.97)], scores were statistically significant (*p* < 0.05) (Model 1). In Model 2, in addition to the confounders in Model 1, GDS and malnutrition were added to the adjustment, and significant associations were observed between anemia with mobility [OR: 1.84 (95% CI: 1.28–2.64)], and stair climbing [OR: 1.36 (95% CI: 1.02–1.81)] (*p* < 0.05). After adding dynapenia to the model, no significant relationship remained between anemia and each item of BADL’s.

**Table 3:** The Barthel activities of daily living results

	Model 1		Model 2	
	OR	*P*	OR	*P*
Feeding	-	-	-	-
Bathing	1.67(1.02–2.74)	0.040	-	-
Dressing	2.80(1.29–5.95)	0.009	2.31(1.04–5.14)	0.04
Toilet use	-	-	-	-
Stairs	1.92(1.15–3.21)	0.012	-	-
Bowel continence	-	-	-	-
Bladder continence	-	-	-	-
Transfer	2.50(1.27–4.87)	0.008	-	-
Mobility	-	-	-	-
Grooming	2.12(1.25–3.62)	0.005	1.86(1.06–3.28)	0.030

OR, Odds ratio; in model-1 *p* values were still significant, even after adjustment for age, DM, HT, CAD and CRF

In Model 2, in addition to the confounders in Model 1, GDS and malnutrition were added to the adjustment

Table 4 shows that after adjusting for age, DM, CHF and CRF, in those with anemia compared to those with no anemia, adverse effects on shopping [OR: 1.23 (95% CI: 1.02–1.43)], food preparation [OR: 1.29 (95% CI: 1.08–1.53)], housekeeping [OR: 1.61 (95% CI: 1.08–2.41)] and mode of transportation [OR: 1.31 (95% CI: 1.10–1.55)] scores were statistically significant (*p* < 0.05) (Model 1). In Model 2, in addition to the confounders in Model 1, GDS and malnutrition were added to the adjustment, and the significant association of anemia with shopping [OR: 1.18 (95% CI: 1.00–1.38)], food preparation [OR: 1.22 (95% CI: 1.02–1.46)] and mode of transportation [OR: 1.25 (95% CI: 1.05–1.49)] remained. After adding dynapenia, there was no significant relationship between anemia and each item of IADL’s.

**Table 4:** Lawton instrumental activities of daily living results

	Model 1		Model 2	
	OR	*P*	OR	*P*
Ability to Use Telephone	1.92(1.22–3.04)	0.005	1.67(1.03–2.70)	0.036
Shopping	1.41(1.08–1.84)	0.011	-	-
Food Preparation	1.44(1.08–1.91)	0.11	-	-
Housekeeping	-	-	-	-
Laundry	-	-	-	-
Mode of Transportation	1.36(1.05–1.75)	0.018	-	-
Responsibility for Own Medications	1.99(1.20–3.30)	0.007	-	-
Ability to Handle Finances	-	-	-	-

OR, Odds ratio; in model-1 *p* values were still significant, even after adjustment DM, HT, CAD and CRF

In Model 2, in addition to the confounders in Model 1, GDS and malnutrition were added to the adjustment

## Discussion

This study investigated the relationship between anemia and basic and instrumental activities of daily living in older male patients. The frequency of DM, CHF, and CRF was higher in those with anemia than those without anemia. Among geriatric syndromes, depression, malnutrition and dynapenia were more common in anemic patients. Dependence on ADLs was higher in older men with anemia than in those without. However, this significant difference did not persist after adjustment for geriatric syndromes, especially dynapenia, that may affect this dependence.

In older adults, anemia has multiple causes and is typically linked to socio-demographic characteristics, lifestyle choices, and chronic conditions including CRF, DM, and CVD, as well as deficiencies in nutrients such as iron, vitamin B12, and folate [31–33]. Nevertheless, in approximately 14–50% of older individuals, the underlying cause of anemia remains unidentified despite extensive examinations [6, 33]. In the current study, we observed anemia (Hb level below 13 g/dl) in 98 (43.9%) of 223 patients. This prevalence value, independent of the etiology, is higher than the prevalence data obtained from other studies. Research findings on anemia prevalence in older men have shown varying results. In one study, Stauder et al. reported that 11% of older males were affected by anemia [34]. Conversely, a different investigation conducted by Awaluddin et al. discovered a higher prevalence rate of 30.7% among older men [35]. The observed differences could be attributed to several factors, including the older average age of our study population, as well as variations in racial and ethnic backgrounds, economic status, food preferences, and coexisting medical conditions. Conversely, research by Chan et al. revealed that anemia was present in 70.5% of older male residents in nursing homes [36]. This high prevalence can be attributed to several factors characteristic of this patient population, including reduced functional abilities, increased occurrence of frailty, and a higher incidence of additional health issues and geriatric conditions such as malnutrition.

In present study, anemia in older men was found to be associated with DM, HT, CAD, and CRF. The relationship between these comorbid diseases and anemia and their possible mechanisms have previously been identified; thus, our results support these findings. A research study conducted by Michalak et al., involving 981 participants, revealed that 24.9% of patients were diagnosed with DM. Additionally, within the same investigation, 23.8% of a subgroup consisting of 105 older male subjects exhibited both anemia and DM [37]. In our study, the rate of patients with anemia and DM was calculated to be 44.9%. Multiple factors contribute to the development of anemia in DM. These include ongoing inflammation, lack of essential nutrients, reduced renin production, coexisting autoimmune conditions, concurrent use of certain HT medications (especially angiotensin II receptor blockers) and antihyperglycemic agents (particularly metformin), alterations in hormone levels, and impaired kidney function [38, 39]. Additionally, men with type 2 DM frequently exhibit obesity, insulin resistance, and reduced testosterone levels, which could potentially elevate their risk of developing anemia [40]. In the presence of CRF, erythropoietin production is impaired, leading to a primary hematologic manifestation characterized by severe hypoproliferative anemia [41]. Consequently, anemia may contribute to the advancement of chronic kidney disease, higher mortality rates, and a diminished quality of life. Since CRF is associated with geriatric syndromes that may negatively affect ADLs in the older adults, we adjusted for its effect in our study [42]. The same may also be true for anemia and CVD. The research conducted by Spazzafumo et al. revealed a link between anemia and increased cardiovascular risk [43]. Therefore, simultaneous treatment of anemia may be an important element of the management of these diseases.

In the present study, we found that the dependence in both basic and instrumental life activities is more common in older men with anemia. This finding is consistent with previous studies. Research conducted by Maraldi et al. revealed that anemic males had a higher likelihood of experiencing impaired physical function and disability, with the risk increasing by a factor of 1.7 [44]. According to Maraldi et al., the inclusion of demographic and lifestyle factors in multivariate analysis only slightly reduced the strength of association. However, when nutritional factors and disease-related variables were considered, they observed a more substantial decrease in the strength of association. In another study, Jiang et al. anemia is associated with poor performance in lower-extremity physical function among Chinese older adults [45]. According to Perna et al. not only anemia, but also iron deficiency can be associated with muscle weakness [46]. Consistent with previous research, this investigation revealed a strong correlation between anemia and dependency. However, when factors such as comorbidities, geriatric depression, and nutritional status were controlled for, the strength of this relationship decreased, though it remained statistically significant. However, we also evaluated the effect of dynapenia on this relationship, and showed that the connection between ADLs and anemia is largely due to dynapenia. Therefore, there is clear importance of anemia-related muscle weakness in an older male patient in maintaining independence in activities of daily living. Notably, when dynapenia and anemia occur together, they are strongly linked to an increased likelihood of death from any cause over a 10-year follow-up period. This association remains significant even after accounting for socioeconomic factors, lifestyle choices, and existing health conditions [47]. Anemia’s impact on reducing oxygen delivery to tissues also impacts musculoskeletal structures, which may explain the diminished strength and muscle performance observed in anemic individuals [48]. These findings regarding anemia’s effect on functional capacity clearly suggest a significant role of dynapenia [18, 49].

One of the strengths of this study is the adequate sample size of patients with anemia. Another strength is that anemia screening and comprehensive geriatric evaluation were performed on the same day. A final strength is that only older men were included in the study; thus, sex-related effects could be excluded. Several limitations of this study should be considered when interpreting findings. Firstly, anemic men were not grouped among themselves according to the severity of anemia and were evaluated only at one visit. Second, erythropoietin levels were not routinely checked in all patients, which is important in deciding whether the anemia is transient or permanent. Another limitation of the study is that functional iron deficiency (transferrin saturation < 20%, ferritin > 100 ng/ml) was not examined in detail. Finally, the study utilized a cross-sectional design and thus direction of the observed association cannot be established it is possible that the relationship is bi-directional, for example, dependence in daily living may lead to malnutrition that subsequently leads to anemia. Future studies utilizing a longitudinal design are now required to elucidate on the direction of the association.

In conclusion, approximately one in every two older men presenting to a Turkish outpatient clinic had anemia. Anemic men were older and had a higher incidence of DM, HT, CRF, malnutrition, geriatric depression and dynapenia. Anemia is associated with dependence in both basic and instrumental activities of daily living. However, dynapenia has an important contribution to this association. Therefore, older men should routinely be tested for anemia, anemia related reduced muscle strength should be investigated and treated in order to prevent dependency on daily life activities.

## Data Availability

No datasets were generated or analysed during the current study.
